# Population genomic analyses of early-phase Atlantic Salmon (*Salmo salar*) domestication/captive breeding

**DOI:** 10.1111/eva.12230

**Published:** 2014-11-20

**Authors:** Hannu Mäkinen, Anti Vasemägi, Philip McGinnity, Tom F Cross, Craig R Primmer

**Affiliations:** 1 Division of Genetics and Physiology, Department of Biology, University of TurkuTurku, Finland; 2 Department of Aquaculture, Estonian University of Life SciencesTartu, Estonia; 3 Aquaculture and Fisheries Development Centre, School of Biological, Earth, and Environmental Sciences, University College CorkCork, Ireland; 4 Marine Institute, FurnaceNewport, Co. Mayo, Ireland

**Keywords:** adaptation, aquaculture, captive populations, ecological genetics, population genetics – empirical

## Abstract

Domestication can have adverse genetic consequences, which may reduce the fitness of individuals once released back into the wild. Many wild Atlantic salmon (*Salmo salar*L.) populations are threatened by anthropogenic influences, and they are supplemented with captively bred fish. The Atlantic salmon is also widely used in selective breeding programs to increase the mean trait values for desired phenotypic traits. We analyzed a genomewide set of SNPs in three domesticated Atlantic salmon strains and their wild conspecifics to identify loci underlying domestication. The genetic differentiation between domesticated strains and wild populations was low (*F*_ST_ < 0.03), and domesticated strains harbored similar levels of genetic diversity compared to their wild conspecifics. Only a few loci showed footprints of selection, and these loci were located in different linkage groups among the different wild population/hatchery strain comparisons. Simulated scenarios indicated that differentiation in quantitative trait loci exceeded that in neutral markers during the early phases of divergence only when the difference in the phenotypic optimum between populations was large. This study indicates that detecting selection using standard approaches in the early phases of domestication might be challenging unless selection is strong and the traits under selection show simple inheritance patterns.

## Introduction

Domestication of various animal and plant species has led to several adaptations to different environmental conditions, and the genetic basis of this variation is well known. For example, the genetic bases of horn size differences in Soay sheep and of morphological differences among dog breeds have been characterized in detail and appear to be based on simple inheritance patterns (Andersson and Georges 2004; Boyko et al. 2010; Johnston et al. 2013). Genomewide scans have also identified several genomic regions associated with artificially selected phenotypic traits (Boyko et al. 2010; Akey et al. 2010; Rubin et al. 2010; Wilkinson et al. 2013). The majority of domestication events appear to date back to the Holocene period (approximately 10 000 years ago) and are likely associated with the establishment of agriculture (Innan and Kim 2004). In contrast, the domestication of fish species, such as Atlantic cod, Atlantic salmon, and Arctic char, commenced more recently, a few tens of generations ago (Hutchings and Fraser 2008), providing an excellent opportunity to investigate the genetics of the early phases of domestication.

Hatchery strains of various fish species are generally maintained for one of the two purposes. First, hatchery-reared individuals are used to supplement endangered natural populations or for re-establishing extinct populations (Frankham 2008; Hutchings and Fraser 2008; Araki et al. 2008; Araki and Schmid 2010). In stock supplementation, wild-caught fish are maintained and bred in hatcheries to produce offspring for later release into the wild. This practice avoids exposing fish to direct anthropogenic selection pressures, but selection may arise as a by-product (inadvertent selection) during the life stages in the artificial environment. Rearing in the hatchery environment can reduce the survival probability or reproductive success of the fish after release into the wild, and these effects can often be detected after only one generation in the artificial environment (Araki et al. 2008; Fraser 2008; Milot et al. 2013). Second, fish can be maintained in captivity throughout all life stages for aquaculture purposes, where they are selected for desired phenotypic traits such as increased growth, flesh quality, parasite resistance, and delayed maturity (Frankham 2008; Araki and Schmid 2010). Selective breeding for increased growth in Atlantic salmon can result in a 15% selection response per generation (Gjeren and Bentsen 1997; Martinez et al. 2013). Both selective breeding and supplementary stocking practices have been shown to induce phenotypic differentiation and can have fitness consequences (Araki et al. 2008; Araki and Schmid 2010). Hybridization between fish originating from supplementary stocking or aquaculture escapees with wild fish may reduce the survival rates and population sizes of wild fish populations and is thus a major concern regarding wild population persistence (Ford and Myers 2008; Fleming et al. 2011). Some practices including minimizing the number of generations spent in captivity and mimicking natural conditions in captive environments have been undertaken to compensate the negative effects of captive breeding, but the results are uncertain (Wilke et al. 2014).

One popular approach to investigate the genetic basis of adaptation to novel environments and domestication is to identify genomic regions showing unusual patterns in allele frequency differentiation (Storz 2005; Begun et al. 2007; Hohenlohe et al. 2010b). In principle, the target of directional or divergent selection and the linked genomic regions should show higher between-population differentiation and lower genetic diversity than the genomewide distribution (Schlötterer 2003; Nielsen 2005). A common statistical approach for detecting such genomic regions is to simulate the distribution of a given test statistic (e.g., *F*_ST_) assuming a neutral model suitable for the data (Beaumont and Rannala 2004). Another approach is to rely on the empirical distribution and apply a genomewide averaging method, such as kernel smoothing to identify genomic regions that show unusual differentiation relative to the genomewide average (Hohenlohe et al. 2010a). The detected outliers and their associated *P*-values should be considered as statistical outliers rather than direct evidence that selection has affected their divergence. Theoretical studies suggest that allele frequency differentiation around the selected site can vary depending on both the type of selective sweep and the genetic background of a given quantitative trait (Hermisson and Pennings 2005; Pennings and Hermisson 2006; Pritchard et al. 2010; Cutter and Payseur 2013). Selection acting on novel adaptive mutations (hard sweeps) is expected to result in a stronger signal around the selected site than when it acts on genetic variation already present in the population (standing variation), commonly known as a soft sweep (Pritchard et al. 2010; Cutter and Payseur 2013). If the genetic background of a given trait consists of many loci with small additive effects, the selection coefficient per locus can be very small and the signal of selection can be weak and difficult to detect (Pritchard et al. 2010). One way to evaluate the power to detect signatures of selection in a given study design is to compare the observed patterns to simulated data if the relevant simulation parameters can be reasonably well approximated (Hoban et al. 2011). This approach has become more feasible due to the development of sophisticated software, enabling the simulation of complex and biologically meaningful population genetic scenarios (Hoban et al. 2011).

Although the fitness effects of captive breeding are relatively well characterized, the genetic basis of adaptation to the hatchery environment is relatively poorly understood. Araki et al. (2008) proposed that deleterious mutation accumulation, inbreeding depression, and domestication selection may all contribute to the fitness decline of hatchery-reared strains. Evidence for domestication/inadvertent selection, that is, selection favoring beneficial alleles or allele combinations in the hatchery environment, is sparse (Karlsson et al. 2011; Vasemägi et al. 2012; Martinez et al. 2013). Earlier studies of Atlantic salmon aiming to detect genomic regions under selection, and potentially underlying domestication used a small number of molecular markers (Vasemägi et al. 2012), focused on describing generic differences between hatchery strains and wild populations (Karlsson et al. 2011) or focused on a narrow genomic region containing a candidate gene for growth (Martinez et al. 2013). Some studies have investigated whether hatchery strains and wild fish populations show differentiation in gene expression levels. These studies have shown that expression differences are heritable and involve a small number of genes showing parallel differential gene expression, suggesting that in some cases, domestication might involve parallel adaptive signals (Roberge et al. 2006; Sauvage et al. 2010). The effect of domestication on quantitative genetic variation in, for example, parasite resistance or swimming behavior has been investigated using common garden experiments (Carlson and Seamons 2008; Solberg et al. 2013; Yáñez et al. 2013; Bellinger et al. 2014). These studies have indicated that there are varying levels of heritability in the studied traits and thus varying potential for selective breeding. Quantitative genetic experiments indicate that the genetic component plays major role relative to the environmental effects (Araki et al. 2007). The genetic basis of economically important traits in domesticated fish species has been intensively studied using a QTL-mapping approach. It appears that most of the genetic variation in quantitative traits is explained by moderate-to-large effect loci, but loci with small effect may have remained undetected due to experimental limitations such as small number of informative loci and/or individuals in mapping families (Yue 2013).

One of the advantages of a generic genome scan approach is that it does not target any specific trait defined *a priori* as in QTL mapping, and thus, it can help to reveal adaptively significant variation that was previously unknown (Schlötterer 2003; Storz 2005). To understand the genetic basis of domestication in Atlantic salmon (*Salmo salar*), we conducted a genomewide scan of single nucleotide polymorphisms to identify regions showing unusual allele frequency differentiation between hatchery strains and their wild conspecifics and which might possibly underlie adaptation to the hatchery environment. This study investigated the same populations as a previous study (Vasemägi et al. 2012), but the number of molecular markers and individuals have been increased to improve genome coverage and provide more reliable estimates of population allele frequencies. Two of the domesticated strains were maintained for supplementary stocking and one for selective breeding purposes. This combination allows us to investigate the potential effects of selection arising inadvertently and due to artificial selection on desired phenotypic traits. Our working hypothesis is that the selection detected in the supplementation stocking strains (R. Burrishoole and R. Vindel/Ume) arises due to inadvertent selection, whereas in the aquaculture strain selection is predominantly due to artificial selection. We also predict that the selection signals are more common in the aquaculture strain than in supplementation stocking strains because selection pressure due to targeted artificial selection is expected to be higher (Biswas and Akey 2006). We identified the genomic regions potentially involved in domestication using standard outlier statistics and tested whether the same genomic regions were identified in different domesticated strains using a kernel-smoothing approach. We simulated population genetic scenarios using a biologically meaningful range of parameters to investigate how selection affects quantitative trait differentiation and whether it can be separated from background differentiation (i.e., neutral marker differentiation) during the early phases of divergence.

## Materials and methods

A total of 256 individuals were analyzed from two supplementation stocking hatchery stains and one selective breeding strain and three wild populations from the same river systems from which the strains originated (Table1). The R. Saint John population belongs to the North American lineage, whereas the R. Burrishoole and R. Vindel/Ume populations belong to the Atlantic and Baltic lineages, respectively (Bourret et al. 2013). The Saint John (New Brunswick, Canada) selective breeding strain originates from wild individuals taken from R. Saint John in 1990. At the time of sampling in 2006, the selective breeding strain had been separated from the wild for approximately five generations, assuming a generation time of 5 years for Atlantic salmon. The Saint John strain has been selectively bred for faster growth and reduced early maturation (Quinton et al. 2005; Vasemägi et al. 2012). The R. Burrishoole (Mayo, Ireland) hatchery strain was initiated in 1960 and supplemented with wild individuals until 1964. A few wild individuals were added between 1970 and 1975, but since that time, the hatchery strain has been managed independently from the wild fish. Therefore, before sampling in 2006, the R. Burrishoole hatchery strain had been isolated from their wild conspecifics for approximately 8–9 generations. The R. Umeälven population went extinct due to the construction of the Norforss dam in the early 1950s, but the R. Vindelälven (a tributary of Umeälven) salmon population survived, and the hatchery strain was established from surviving individuals. Each year, the majority of migrating salmon are caught in front of the Norforss dam, and only fish with intact adipose fins (i.e., of wild origin) are released upstream of the Norforss dam to R. Umeälven. Some of these latter individuals were taken as a wild reference population. This practice began in 1974, and thus, the hatchery strain had been isolated from the wild for approximately 5–6 generations at the time of sampling.

**Table 1:** SNP data filtering statistics. Numbers indicate the number of SNPs retained after each filtering step

	Sample size	Missing data/locus (>30%)	Alleles (0,1,3)	MAF (<5%)	HW test (*P* < 0.001)	Common	Mapped
Burrishoole wild	44	7760	7694	6357	5242	4733	4198
Burrishoole dom	52	7867	6948	6191	5749		
Vindel/Ume wild	48	6582	6372	5499	4359	4039	3547
Vindel/Ume dom	48	6532	6295	5497	4368		
Saint John wild	40	6789	6339	4785	3694	2797	1750
Saint John dom	24	6712	5600	4368	3406		

dom, domesticated.

### SNP genotyping and filtering

DNA was extracted from fin clips or muscle tissue using a salt-extraction method (Aljanabi and Martinez 1997). A salmon SNP chip described in Lien et al. (2011) was used for genotyping. Briefly, the SNP discovery panel was based on expressed sequence tag (EST) alignments and random genomic libraries constructed from eight individuals and sequenced with 454 technologies. The EST alignments were derived from several individuals of both the McConnell strain (Canada) and the Aquagen breeding company (Norway) strain. The initial data set contained 15 164 genotypes before quality filtering. The quality filtering procedure and the number of loci retained after each step are reported in Table1. The filtering steps included removal of those loci with completely missing data (0 alleles), monomorphic loci (one allele), or potentially duplicated loci (three alleles). Further filtering steps included removing those loci with 30% missing data, <5% minor allele frequency (MAF) and loci deviating from Hardy–Weinberg expectations (Fisher's exact test, 0.001 significance level). The Hardy–Weinberg test included loci showing both a deficit and an excess of heterozygosity. It has been shown that uninformative polymorphisms, that is, alleles segregating at low frequencies among populations, may affect the baseline differentiation and thus the outcome of *F*_ST_ outlier tests (Roesti et al. 2012). Applying a universal 5% MAF threshold may filter out loci almost fixed for the alternative alleles, and thus, some selection signals could be lost. Without a MAF threshold, the number of outlier loci remained the same in all comparisons, suggesting that 5% threshold was appropriate for our data. These filtering steps were conducted for each population separately, and a common SNP data set was combined for each wild population/hatchery strain pair. After filtering, 4733, 4039, and 2797 polymorphic loci were retained for the R. Burrishoole, R. Ume/Vindelälven, and R. Saint John, respectively. The location of the polymorphic loci was assigned according to the Atlantic salmon linkage map (Lien et al. 2011), and 4198, 3547, and 1750 loci were retained for R. Burrishoole, R. Ume/Vindelälven, and R. Saint John wild population/hatchery strain pairs, respectively. The mapped data sets were used for the kernel-smoothing analyses only, as this type of analysis requires that the genomic position of each locus be assigned. There is considerable uncertainty in the map positions of the R. Saint John population because it belongs to the North American salmon lineage, where chromosomal re-arrangements relative to European origin *S. salar* have been reported (Lubieniecki et al. 2010). Therefore, the reported positions should be considered as tentative.

### Estimation of basic population genetic parameters

Observed and expected heterozygosities were estimated according to Nei (1987). Deviations from Hardy–Weinberg expectations (*F*_IS_) for each population were estimated according to Weir and Cockerham (1984). Genetic differentiation between wild and domesticated strains was estimated using Weir and Cockerham's estimator of *F*_ST_ (Weir and Cockerham 1984), and the 95% confidence intervals were estimated by bootstrapping (1000 replicates) over loci. These estimates were calculated as implemented in the R package HierFstat (Goudet 2005). Population-specific *F*_ST_ estimates were calculated according to Weir and Hill (2002), and a Bayesian estimator of *F*_ST_ was also calculated using the BayeScan 2.1 package (Foll and Gaggiotti 2008).

### Detecting loci and genomic regions under selection

Two approaches were used to detect candidate loci for selection based on either the empirical distribution of standardized heterozygosity (Kauer et al. 2003) or the simulated distribution of *F*_ST_ (Foll and Gaggiotti 2008). Standardized heterozygosity (LnRH) for each locus was estimated according to formula 2 of Kauer et al. (2003). The distribution of LnRH should follow a normal distribution, and the loci at the tails of the distribution are considered to harbor significantly lower genetic diversity than the genomewide average. Thresholds of −2.58 and 2.58, corresponding to the *P*-value of 0.01, were used to detect loci deviating from genomewide average. Simulation studies have shown that the LnRH should follow a normal distribution under a wide range of population genetic conditions, and thus, the normal distribution should in theory apply to our study populations (Kauer et al. 2003). The LnRH statistics allows only pairwise comparisons, and therefore, LnRH was estimated for each wild/domesticated pairwise comparison. The distribution of *F*_ST_ was simulated, assuming that subpopulations were derived from a common gene pool and that the migration rates between the common gene pool and subpopulations can vary. If this expectation holds, neutral allele frequencies can be modeled according to the multinomial Dirichlet sampling distribution (Beaumont 2005; Foll and Gaggiotti 2008). It has been shown that if the subpopulations do not share a common gene pool, for example, if they belong to different evolutionary lineages, the distribution of *F*_ST_ assuming multinomial sampling can be biased. This situation may increase the number of false positives (Excoffier et al. 2009). Therefore, simulations were carried out using pairwise comparisons for each wild/domesticated pair, as the study populations belong to distinct evolutionary lineages (Bourret et al. 2013). In practice, different types of selection can be inferred from the parameter *F*_ST_ by separating the effects that are common to all loci (population effects, beta) from those that have locus-specific effects (alpha). Selection is expected to affect locus-specific differentiation, whereas genetic drift affects all loci approximately equally. Therefore, the posterior distribution of alpha can be informative with respect to different forms of selection. If the posterior distribution of alpha deviates from zero, or 95% of the distribution is positive, diversifying selection may have affected the differentiation of a given locus (high-*F*_ST_ outlier). If the opposite pattern is observed, that is, the distribution of alpha is negative, balancing selection can be inferred (low *F*_ST_ outlier). Simulation of the *F*_ST_ distribution was carried out using BayeScan 2.1 software with a burn-in period of 50 000 iterations and a thinning interval of 10, resulting in a total of 100 000 iterations. Twenty short runs of 5000 iterations were used to adjust the acceptance rates for each model parameter before the actual run. Five thousand simulated samples from the stationary phase of the MCMC were used to estimate the posterior distributions of the model parameters. The posterior odds for the neutral model were set at ten times higher than that of the model including selection.

### Detection of clusters of outlier loci by kernel smoothing

The genomic distribution of genetic divergence (*F*_ST_) and diversity (LnRH) was further analyzed by a kernel-smoothing approach. The aim was to identify whether the outlier loci identified by BayeScan or LnRH analyses cluster in certain genomic positions and whether the clusters are located at approximately the same genomic positions in the different domesticated strain/wild population comparisons, indicating parallel signals of adaptation. The kernel-smoothing approach is also likely to separate single outlier loci from genomic regions containing multiple outlier loci and may help to exclude false-positive loci. An automatic bandwidth selection for fitting the kernel-smoothed regression line was used to take into account the variation in genetic distances (in cM) between SNP loci. The intermarker distances at the beginning of linkage group 12 were large and frequently caused crashes of the kernel-smoothing algorithm. Therefore, marker rs159405770, located at position 8.4 cM, was excluded from the final analyses of chromosome 12 in all comparisons. A permutation test (10 000 replicates) was used to compare the simulated and observed distributions of kernel-smoothed *F*_ST_ and LnRH. Genomic regions with *P*-values <0.001 were considered as deviating from the genomewide average. Kernel smoothing was conducted using the R package KernSmooth 2.23 and implemented with custom-made scripts.

### Linkage disequilibrium decay

Theory suggests that hard and soft selective sweeps can affect patterns of LD around the selected locus and that these can be separated from neutrally evolving regions (Pokalyuk 2012). Hard selective sweeps are expected to increase LD over larger distances than soft sweeps (Pritchard et al. 2010; Cutter and Payseur 2013). Explorative linkage disequilibrium (LD) analyses were conducted in those linkage groups identified as containing outlier loci in the BayeScan analysis. Two estimators of LD (r2 and D') were used, and a heat map was constructed to visualize local patterns of LD, implemented in the R package LD heat map (Shin et al. 2006). In addition, the decay of LD with genetic distance was measured by intermarker LD estimates (r2 and D') and by fitting regression lines, following Hill and Weir (1988). The decay of LD was estimated by calculating the genetic distance at which half of the LD signal had decayed.

### Simulated data sets

To understand quantitative trait and neutral marker divergence, hypothetical population genetic scenarios were simulated using parameters resembling the wild salmon population/hatchery strain differentiation. The main objective was to simulate the effects of different genetic backgrounds and selection pressures on quantitative trait (QTL) differentiation relative to the neutral marker divergence during the early phases of domestication. This should shed light on the conditions and time scale when QTL divergence exceeds that of the neutral marker divergence and thus the possibility of detecting selection. Simulation parameters are summarized in Table2, but a brief justification of model parameters is given below. All simulations included two populations of 200 individuals each, random mating, and no mutation nor gene flow for both quantitative traits and neutral loci. The assumed effective population size was based on the average estimate across the study populations as reported by Vasemägi et al. (2012). These Ne estimates were calculated according to the formula Ne = *t*/(2*F*_ST_), where *F*_ST_ is the differentiation between hatchery strain and wild population and *t* is the number of generations since the split. The effective population sizes were 330, 65, and 238 for R. Burrishoole, R. Vindel/Ume, and R. St. John, respectively. Prior information on the domesticated populations suggests an absence of gene flow between the domesticated strains and their wild conspecifics since the initiation of the hatchery practices (Vasemägi et al. 2012). Given the short divergence time, it was assumed that no mutations had occurred after the domesticated–wild split. In other words, the diversity in QTLs exists in the populations, and therefore, selection operates on standing genetic variation in all simulations. For simplicity, all neutral markers and QTLs were assumed to be located in linkage group 11. One quantitative trait was simulated as coded by either five or 20 loci (QTLs), each harboring two alleles. The QTLs were assumed to be located in linkage group 11 at positions 5, 20, 40, 50, and 70 (five loci) and 2, 5, 10, 15, 20, 27, 30, 35, 40, 45, 47, 50, 55, 60, 65, 67, 70, 75, and 80 (20 loci), as measured in centimorgans (cM). The variance in allele effects was set to vary from 0.1 to 1, and the effects were drawn randomly from a normal distribution with mean of zero. The selection intensity remained constant throughout all simulations, but the phenotypic optima varied between populations. It was assumed that one population had an optimum at zero, while the other had an optimum varying between 0.1, 0.5, and 5, resembling a case where selection acts only in the hatchery strain. Neutral marker evolution was based on a similar marker number (177), and the same map positions as in linkage group 11 in R. Burrishoole. Each neutral marker contained two alleles to resemble the SNP data. All simulations were run for 100 generations and 1000 replicates, and the neutral and quantitative trait loci were simulated in the same run. A control simulation without selection was performed for each simulation to estimate the potential increase in background divergence due to selection. The simulated data sets were recorded every second generation, and *F*_ST_ and its standard deviation were used to estimate divergence in the neutral loci and QTLs. All simulations were conducted as implemented in the software quantiNemo (Neuenschwander et al. 2008).

**Table 2:** Parameters used in the simulations of quantitative trait evolution in the domesticated strains. Other relevant parameters are described in the Materials and methods section

	Sim_A	Sim_B	Sim_C	Sim_D	Sim_E	Sim_F	Sim_G	Sim_H	Sim_I	Sim_J	Sim_K	Sim_L
No. of quantitative traits	1	1	1	1	1	1	1	1	1	1	1	1
No. of QTL	5	5	5	5	20	20	20	20	5	5	20	20
No of alleles	2	2	2	2	2	2	2	2	2	2	2	2
Selection optimum	0.1	0.5	0.1	0.5	0.1	0.5	0.1	0.5	5	5	5	5
Selection intensity	1	1	1	1	1	1	1	1	1	1	1	1
Variance of allele effects	0.1	0.1	1	1	0.1	0.1	1	1	0.1	1	0.1	1

## Results

### Basic population genetic parameters

After the filtering steps, 4733 (R. Burrishoole), 4039 (R. Vindel/Umeå), and 2797 (R. Saint John) SNP loci were retained, corresponding to approximately one SNP per 0.50, 0.59, and 0.85 cM, respectively (Table1). The lower number of polymorphic loci observed in the North American populations is consistent with earlier results, showing that lower diversity is typical for North American Atlantic salmon (Bourret et al. 2013). In addition, ascertainment bias may have contributed to the observed lower diversity in North American populations, as the SNP discovery panel consisted primarily of European Atlantic salmon individuals. The R. Burrishoole wild population harbored slightly higher mean observed and expected heterozygosities than the hatchery strain (Table3, Wilcoxon signed rank test, *H*_O_ *P* = 9.7 × 10^−6^; *H*_E_ *P* = 9 × 10^−6^). The expected and observed heterozygosity in the R. Vindel/Ume wild population was higher than in the hatchery strain, but only the difference in observed heterozygosity was statistically significant (Table3, Wilcoxon signed rank test, *P* = 0.03). In contrast, the R. Saint John hatchery strain showed higher expected and observed heterozygosity; however, only the difference in expected heterozygosity was statistically significant (Table3, Wilcoxon signed rank test, *P* = 0.008). Only small deviations from Hardy–Weinberg expectations were detected at the population level, indicating slight heterozygote excess. Overall, genetic differentiation between the hatchery strains and wild populations was relatively low. The lowest *F*_ST_ was observed between the R. Vindel/Ume hatchery strain and wild population (mean 0.0069, 95% CI, 0.0058–0.0074), whereas the R. Burrishoole (mean 0.0149, 95% CI, 0.0138–0.0159) and R. Saint John (mean 0.0314, 95% CI, 0.0287–0.0341) showed higher levels of differentiation. In general, most of the genetic differentiation was associated with the hatchery strains, as indicated by the higher population-specific *F*_ST_ estimates. A reversed pattern was observed in R. Saint John comparison (Table4).

**Table 3:** Estimates of basic population genetic parameters in the study populations

	*H*_o_	*H*_e_	*F*_IS_	*F*_ST_
Burrishoole wild	0.383	0.381	−0.0069 (−0.0106 to 0.0001)	0.0148 (0.0138–0.0159)
Burrishoole dom	0.370	0.370	−0.0037 (−0.0068 to 0.0033)	
Vindel/Ume wild	0.372	0.363	−0.0245 (−0.0298 to 0.0183)	0.0066 (0.0058–0.0074)
Vindel/Ume dom	0.365	0.358	−0.0205 (−0.0241 to 0.0137)	
Saint John wild	0.333	0.320	−0.0335 (−0.0472 to 0.0302)	0.0314 (0.0287–0.0341)
Saint John dom	0.343	0.328	−0.0413 (−0.0555 to 0.0348)	

### Putative outlier loci and genomic regions under divergent selection

**Figure 1:**
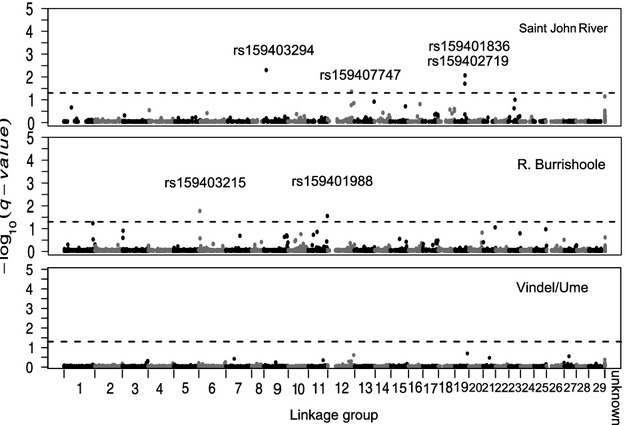
Manhattan plots showing the results of the BayeScan *F*_ST_ outlier tests applied across the domesticated strain/wild population pairs. Salmon linkage groups where the SNP loci were mapped are indicated on the *x*-axis. ‘Unknown’ refers to SNPs that were not mapped on the Lien et al. (2011) linkage map. The log^−10^ of q-values indicating the probability of the locus being a outlier are on the *y*-axis. The dashed red line indicates the q-value threshold at 0.05.

Overall, very few to no outlier loci were detected in the BayeScan analyses. The largest number of *F*_ST_ outlier loci was detected in the R. Saint John domesticated/wild comparison, where four loci located in linkage groups 9, 12, and 19 showed signals of divergent selection at the 5% false discovery rate (Fig.1). In the R. Burrishoole comparison, two loci located in linkage groups 6 and 11 were detected as high-*F*_ST_ outliers (Fig.1). No outlier loci were detected in the R. Vindel/Ume comparison (Fig.1). *LnRH* tests revealed a larger number of putative outlier loci. In the R. Burrishoole comparisons, 43 and 72 loci in the domesticated strain and wild population, respectively, had LnRH estimates exceeding ±2.58 (i.e., *P*-value <0.01) (Fig.3). Thirty-nine loci in the domesticated R. Vindel/Ume strain and 43 in the wild population were detected as outliers based on the aforementioned thresholds (Fig.3). In the R. Saint John salmon, the domesticated strain had 33 outlier loci and the wild population had 20 (Fig.3). At a significance level of 0.01, 47, 40, and 28, loci are expected to have *P*-values less than or equal to 0.01 for R. Burrishoole, R. Vindel/Ume, and R. Saint John, respectively. Thus, some of the LnRH outlier loci are presumably false positives.

**Table 4:** Population-specific *F*_ST_ estimates and their 95% confidence intervals (Weir and Hill 2002, WH 2002) or 95% posterior density intervals (BayeScan)

	*F*_ST_ (WH 2002)	*F*_ST_ (BayeScan)
Burrishoole wild	−0.0121	0.0026 (0.0010–0.0045)
Burrishoole dom	0.0120	0.0237 (0.0211–0.0259)
Vindel/Ume wild	−0.0114	0.0024 (0.0008–0.0041)
Vindel/Ume dom	0.0022	0.0082 (0.0061–0.0102)
Saint John wild	0.0296	0.0302 (0.0249–0.0355)
Saint John dom	−0.0027	0.0136 (0.0085–0.0192)

**Figure 2:**
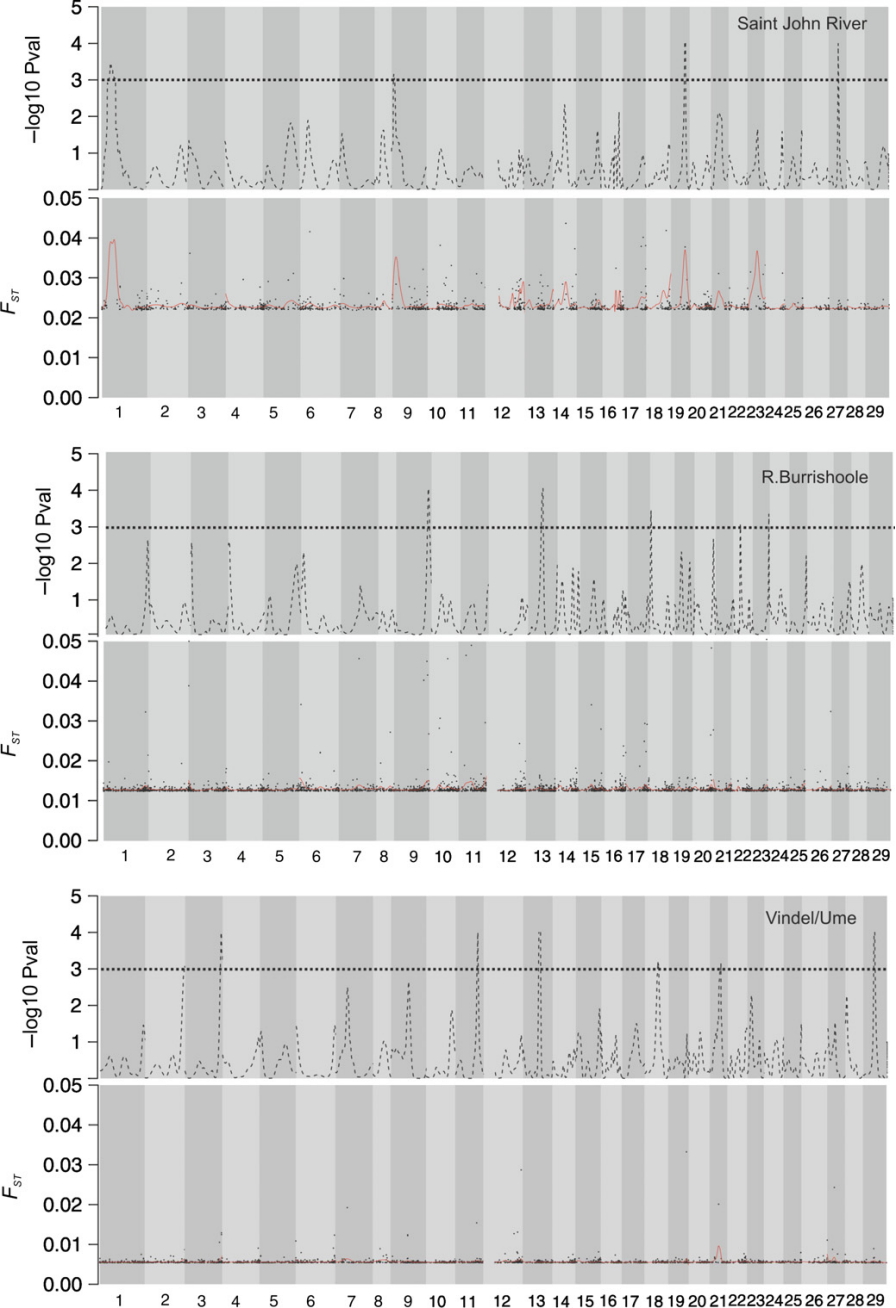
Kernel-smoothing results for *F*_ST_. In each panel, the lower figure shows the kernel-smoothed *F*_ST_ (red line) and the point estimates (black circles). The upper panels show the distribution of the permuted *P*-value, and the dashed black line indicates the threshold for the significance level (*P*-value ≥0.001).

Permutation of Kernel-smoothed *F*_ST_ values revealed four genomic regions in linkage groups 9, 13, 18, and 23 that deviated from the genomewide average (*P*-value <0.001) in the R. Burrishoole domesticated/wild comparison (Fig.2). Three of these regions were located at the ends of the linkage groups (9, 18 and 23), and one was located in the middle of linkage group 13. In the R. Vindel/Ume comparison, seven genomic regions showed elevated divergence (*P*-value <0.001) and these were located in linkage groups 2, 3, 11, 13, 18, 21, and 29 (Fig.2). Two of the identified regions were observed at the ends of linkage groups (2, 3), and the remainder (11, 13, 18, 21 and 29) were positioned in the middle of the linkage groups (Fig.2). In the R. Saint John comparison, four genomic regions differed significantly (*P* < 0.001) from the genomewide average differentiation. These were located in linkage groups 1, 9, 19, and 27; three of them (1, 19 and 27) were located in the middle of the linkage groups, and one was located at the end of the linkage group 9 (Fig.2).

**Figure 3:**
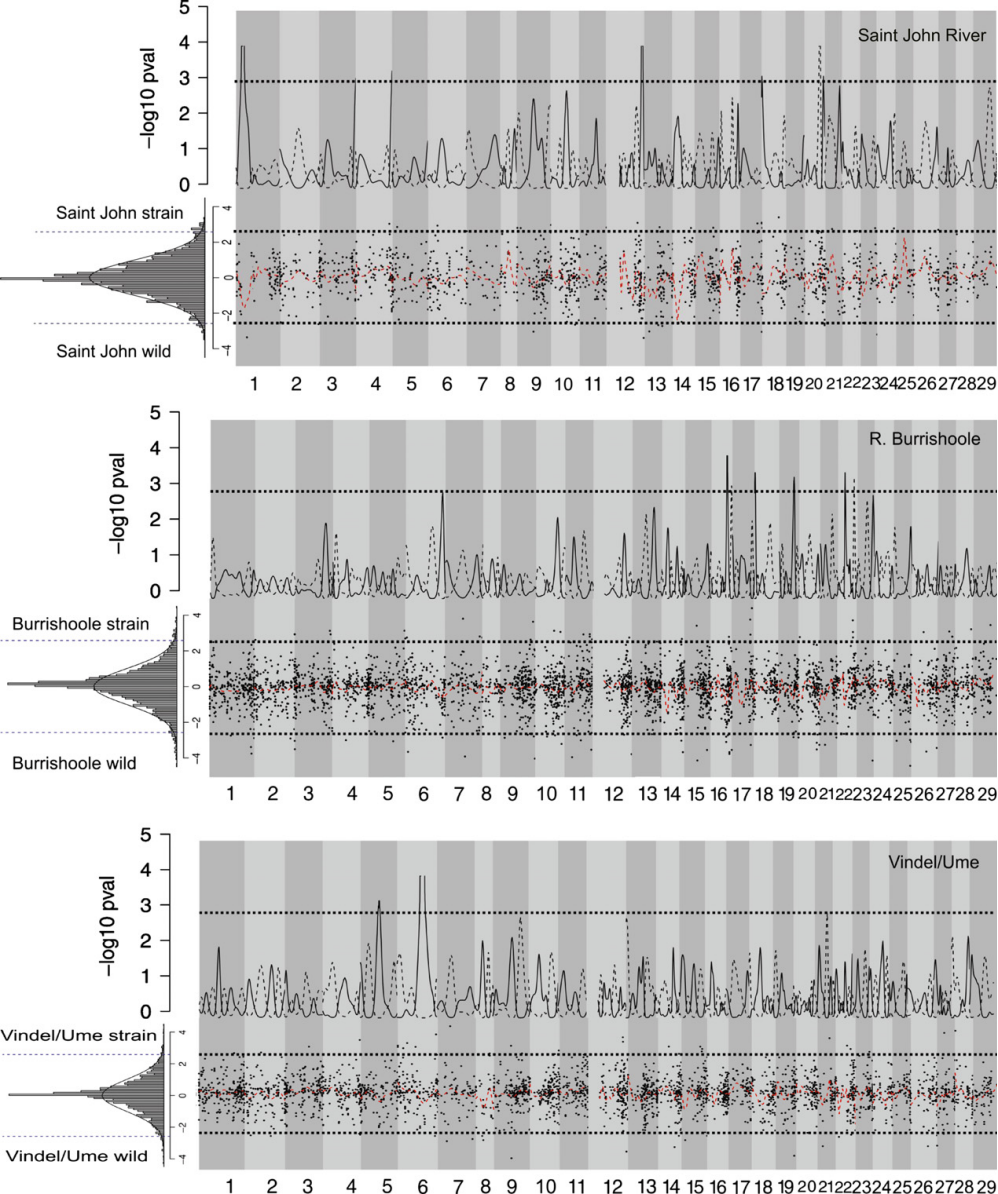
Kernel-smoothing results for LnRH. In the inset map, the histogram shows the distribution of LnRH estimates in 0.1 bins and the solid line indicates the fitted normal distribution. In the lower panel, the red line indicates the kernel-smoothed LnRH, and the solid circles indicate the point estimates. Dashed lines show the significance thresholds of 2.58 and −2.58, corresponding to a *P*-value of 0.01. The upper panel shows the distribution of the permuted *P*-value and the dashed line indicates *P*-value threshold at 0.001.

Permutation of Kernel-smoothed LnRH estimates revealed genomic regions with reduced genetic diversity (LnRH) in linkage groups 16, 18, 19, 22, and 16 and 22 for the wild population and hatchery strain in R. Burrishoole populations, respectively (*P*-value <0.001) (Fig.3). In the R. Vindel/Ume wild population, two genomic regions in linkage groups 5 and 6 had lower diversity than the genomewide average, but no genomic regions in the domesticated strain showed lower genetic diversity than the genomewide average (Fig.3). Four genomic regions in linkage groups 1, 12, 18, and 21 in the wild R. Saint John wild population and one region located in linkage group 20 in the domesticated strain had lower genetic diversity as compared to the genomewide average (Fig.3).

Overall, the overlap in genomic positions containing outlier loci or ‘high-*F*_ST_ genomic regions’ between populations and outlier statistics was relatively small. Two genomic regions (linkage groups 9 and 19) in the R. Saint John comparison contained outlier loci identified by both the BayeScan analysis and the kernel-smoothing approach. The kernel smoothing of *F*_ST_ identified one genomic region located in the same position (linkage group 13) in the R. Burrishoole and R. Vindel/Ume comparisons. Similarly, overlap between LnRH and *F*_ST_ in the kernel-smoothing approach was found only in the R. Saint John comparison (linkage group 1) and the R. Burrishoole comparison (linkage group 18).

The patterns of linkage disequilibrium showed fast decay over short genetic distances. The half LD-decay distances were larger in the R. Burrishoole (0.6 cM) and R. Saint John (2.2 cM) domesticated strains than in the wild populations (Burrishoole, 0.2 cM; Saint John, 0.9 cM). The Vindel/Ume hatchery strain and wild population had the same LD half-decay distance (0.4 cM). Visual inspection of the LD heat maps indicated a lack of elevated LD around the outlier loci detected in the BayeScan analyses (Figures S1 and S2).

### Simulated data sets

**Figure 4:**
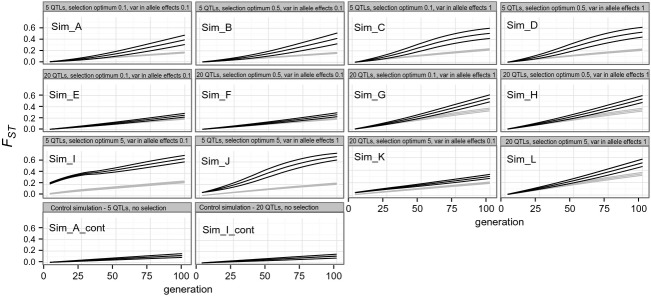
Simulation results for quantitative traits and neutral markers. In each plot, the mean and standard deviation for QTL differentiation as measured with *F*_ST_ are shown in black; for the neutral markers, these are shown in gray. Control simulations are shown for cases A and I only as all simulations produced similar patterns. The number of generations is indicated on the *x*-axis and *F*_ST_ on the *y*-axis.

Most of the simulated data sets showed a linear increase in divergence (*F*_ST_) for both neutral markers and QTLs over generations. In addition, in the majority of the simulated data sets, quantitative trait divergence did not exceed the divergence in neutral markers within the first 10 generations (Fig.4, Sim_A–Sim_H). This was the case when the number of loci coding for quantitative traits was set to either five or 20 and/or when the selection optima varied between 0.1 and 0.5. In addition, changing the variance of allele effects from 0.1 to 1 had no apparent effect on divergence within the first 10 generations (Fig.4, Sim_A–Sim_H). In contrast, after approximately 50 generations, the *F*_ST_ for QTLs exceeded the neutral marker divergence (i.e., their standard deviations did not overlap). A steep increase in *F*_ST_ for QTLs in the first 10 generations was observed only in the simulation with a phenotypic optimum set to 5 (i.e., a 50-fold increase), variance for allele effects set to 0.1 or 1, and a number of loci coding for quantitative traits of five (Fig.4, Sim_I, Sim_J). When the number of QTLs was set to 20 and the variance of allele effects was small (0.1), the simulated distribution also exceeded the neutral marker divergence, but the increase was linear (Sim_K). When the variance of allele effects was 10 times higher (1), the divergence of QTLs and neutral markers overlapped for approximately the first 50 generations (Sim_L). Control simulations, that is, conducted in the absence of selection, indicated that QTL and neutral marker divergence overlapped across the simulated generations but that the overall differentiation was lower than when selection was included in the model (Fig.4, Sim_A_cont, Sim_I_cont).

## Discussion

Our results suggest that the footprints of selection are rare in Atlantic salmon after approximately 10 generations of domestication/captive breeding. The few outlier loci and genomic regions identified in this study were not supported by different statistical approaches. The detection of few outlier loci is somewhat unexpected, considering that domesticated fish strains show differentiation in fitness-related traits and that artificial environment induces behavioral and morphological responses in only a few generations (Araki and Schmid 2010). In addition, selection responses in selective breeding programs can be substantial, indicating that there is genetic potential for large shifts in mean trait values (Martinez et al. 2006). However, several issues regarding the data in the present study and the genetic background of quantitative traits could potentially explain the observed low incidence of footprints of selection.

Empirical studies in wild populations of various taxa employing a few hundred molecular markers have identified 5–10% of loci as outliers (Nosil et al. 2009). In addition, footprints of natural selection are frequently detected in wild salmon populations (Bourret et al. 2013; Perrier et al. 2013; Zueva et al. 2014). Experimental evolution, or ‘evolve and sequence’, studies in *D. melanogaster* suggest that temperature or parasite treatments may induce major allele frequency shifts at hundreds of loci after only a few generations (Orozco-terWengel et al. 2012; Jalvingh et al. 2014). In experimental studies, multiple populations are exposed to similar selection pressures and whole genomes are sequenced, providing high-density maps of genomic events. In contrast, genome scans for selection studies in domesticated fish strains are rare, and thus, comparisons are difficult to conduct. In zebrafish (*Danio rerio*), comparison of laboratory strains to wild populations revealed 59 of 1832 (3.1%) loci as indicating domestication selection (Whiteley et al. 2011). However, the domestication history of zebrafish laboratory strains is longer (*c*. 30 generations) than in *S. salar* and may involve bottlenecks, which might lead to false-positive signals of selection (Whiteley et al. 2011). A previous study (Vasemägi et al. 2012) of the same populations as studied here, based on approximately 350 SNPs and microsatellites, identified ten outlier loci/regions supported by multiple outlier statistics, a number comparable to that identified in the present study. A higher density of molecular markers is expected to increase the linear coverage of the genome and should therefore lead to a higher probability of detecting genomic events, such as footprints of selection (Davey et al. 2011). However, at least in the present study, increasing the number of markers by approximately 10-fold did not increase the resolution or result in a higher proportion of outlier loci relative to the previous study (Vasemägi et al. 2012). LD analyses indicated that linkage disequilibrium decayed relatively rapidly in the salmon genome (half LD distance: 0.2–2.2 cM). Assuming that (i) a footprint of selection can be detected in this window, (ii) markers are evenly spaced within the genome, and (iii) the total salmon map length is approximately 2500 cM, an estimate of the total number of markers needed to cover the entire genome can be calculated. An average of 5500 (1136–6220, except for the R. Burrishoole wild population estimate of 12 500) molecular markers would be needed to efficiently map the salmon genome, a number comparable to that used in the present study. However, our marker set was not evenly spaced, and most markers were located at the ends of linkage groups (Figure S2). A previous study using microsatellites tightly linked to a QTL affecting growth identified a footprint of selection in salmon strains experiencing strong artificial selection (Martinez et al. 2013). This result is in agreement with earlier empirical and theoretical studies, suggesting that tight linkage to the target of selection is needed to detect footprints of natural selection (Storz 2005). In conclusion, the uneven distribution and relatively low density of SNP markers employed in the present study may have contributed to the low number of outlier loci detected.

The simulation results suggest that quantitative trait architecture, the strength of selection, and the short time interval since the initiation of the captive breeding may all contribute to a reduced probability of detecting selection. If the phenotypic optimum between populations is moderate, the differentiation in QTLs does not exceed that of neutral markers within approximately the first 50 generations. *F*_ST_ in QTLs can only exceed the neutral marker divergence in the first 10 generations when the difference in optima is very large (i.e., 50-fold), the quantitative trait is coded by five loci, and the variance for allele effects is small (0.1). This finding suggests that the probability of detecting footprints of selection in the early stages of domestication may be low if the difference in phenotypic optima is moderate. In contrast, the *F*_ST_ for QTLs exceeded that of the neutral markers after 50 generations, suggesting that most of the parameter combinations will eventually lead to increased divergence in QTLs, but that this divergence would require more generations than typically observed in domesticated fish strains (i.e., less than ten generations). Our simulation results are also in accordance with an earlier simulation study that evaluated the importance of a set of population genetic parameters in detecting *F*_ST_ outliers in domesticated salmon strains (Karlsson and Moen 2010). The authors demonstrated that the power to detect selection at a single locus depends primarily on the number of generations since domestication, the strength of selection, and the number of populations included in the study. Additionally, a simulation study evaluating the power to detect selection in ‘evolve and sequencing’ studies indicated that large population size, a large number of populations, high genetic diversity, and a large selection coefficient facilitate selection mapping (Baldwin-Brown et al. 2014).

It has been suggested that if the genetic background of a given trait consists of many loci with small additive effects, the selection coefficient for each locus is very small that a large number of generations are needed to accumulate a footprint of selection (Pritchard et al. 2010; Pritchard and Di Rienzo 2010). This suggestion is supported by our simulation results, which indicated that the rate of differentiation in a QTL coded by 20 loci is about five times slower than that of a QTL with five loci. We observed considerable overlap between neutral marker and QTL divergence during first 50 generations in the 20 loci case, whereas in the case of QTL coded by five loci, overlap was observed within the first 10 generations. It should also be noted that the results of the simulated scenarios depend partly on the assumed effective population size such that smaller or higher Ne would result in higher or lower background differentiation. This in turn could affect the conditions where neutral (background) differentiation can be separated from the QTL divergence in populations with low Ne. In cattle breeds, several commercially important traits with different genetic backgrounds have been intensively selected, but clear selection footprints have been detected only in those traits with monogenic inheritance. Despite intensive selection of complex traits (i.e., involving many loci of small effects) such as milk production, the associated genomic regions show no signals of classical selective sweeps (Kemper et al. 2014). Additionally, the type of selective sweep affects the signal of selection at the genomic level. Selection acting on standing genetic variation typically leads to a weaker signal than that operating on novel mutations (Hermisson and Pennings 2005; Messer and Petrov 2013). It is reasonable to assume that novel mutations play a minor role in adaptation to the hatchery environment, as divergence times are short and mutation rates are typically low (in the order of 10^−8^–10^−9^). In conclusion, the short time period of Atlantic salmon domestication, the presumably polygenic background of most quantitative traits, selection operating on standing genetic variation, and the relatively low and uneven density of SNPs in the present study may have contributed the low observed prevalence of robust footprints of selection in our study system.

Gene expression studies in domesticated Atlantic salmon and Arctic charr (*Salvelinus alpinus*) strains have shown that domestication may involve the same genes in different strains (Roberge et al. 2006; Sauvage et al. 2010). There is also evidence that the same genomic regions show divergent selection in different wild Atlantic salmon populations, indicating that some adaptive traits show signals of parallel evolution (Perrier et al. 2013). We found little evidence of parallel signals of adaptation in our study populations. The few outlier loci identified by the Bayesian method were located in different linkage groups in the R. Burrishoole and R. Saint John salmon comparisons. In addition, the kernel-smoothing approach supported the view that parallel selection signals are rare in the analyzed populations/strains. A ‘high-*F*_ST_ peak’ approximately in the same genomic location was observed only in linkage groups 13 and 18, but the peaks were detected in only two of the three comparisons. However, this result should be interpreted with caution as our study strains have experienced different selection pressures (inadvertent versus intentional selection), the density of SNPs differed between comparisons, and the marker positions may differ between the European and North American *S. salar* due to chromosomal re-arrangements. Alternative mechanisms, such as differential gene expression, can explain rapid responses to changing environmental conditions (López-Maury et al. 2008). It has been suggested that environmental stimuli may induce epigenetic changes, which in turn can affect gene expression patterns in novel environments (Li and Leatherland 2013). It is thus possible that alternative molecular mechanisms, such as gene expression tuning (Roberge et al. 2006) regulated with epigenetic modifications and coupled with classical evolutionary adaptation, could explain the early phases of Atlantic salmon domestication and the apparent fitness declines (Araki et al. 2008).

We also found very little congruence between the outlier detection methods, which is a common observation in genome scans for selection studies. Akey (2009) found little overlap among 21 studies of signals of positive selection in humans. The majority of identified genomic regions and genes were supported by only one study (Akey 2009). Simulation studies have identified considerable variation in false-positive and false-negative rates among different *F*_ST_ outlier methods, with BayeScan having the lowest false-positive rate (Excoffier et al. 2009; Narum and Hess 2011). Some authors have suggested that only those outliers supported by multiple statistics should be considered as the most promising candidates. Using this approach, LnRH and *F*_ST_ supported the outlier status of two genomic regions in a kernel-smoothing approach in two comparisons, and these regions may therefore be considered candidates for further studies. However, kernel-smoothing results should be interpreted with some caution. Choosing the correct window and increment sizes is challenging and can lead to the identification of false peaks (Schmid and Yang 2008; Hofer et al. 2012).

The loss of genetic diversity and inbreeding is a major concern of captive breeding programs (Frankham 2008; Araki et al. 2008; Araki and Schmid 2010). Inbreeding has also been suggested as explaining the fitness reduction in hatchery populations (Araki et al. 2008). Only a small number of individuals can be maintained in the hatchery environment, constituting only a small fraction of the genetic diversity observed in the wild. The loss of genetic diversity has been observed in Atlantic salmon domesticated strains and in other domesticated fish species as well (Araki et al. 2008). The loss of genetic diversity (*F*) since the initiation of the hatchery strains can be estimated assuming that wild populations represent the diversity in the founding hatchery strain and can be estimated according to formula *F *= (*H*_wild_−*H*_domesticated_)/*H*_wild_, where *H* refers to the expected genetic diversity. The estimated values of *F* for R. Burrishoole, Vindel/Ume, and R. Saint John were 0.0288, 0.0137, and −0.025, respectively. This suggests that reductions in genetic diversity were small within the first 5–8 generations of hatchery practice, and the study strains therefore do not appear to be particularly inbred. In steelhead (*Oncorhynchus mykiss*), inbreeding has been shown to explain only 1–4% of the fitness loss in captive-born fish (Christie et al. 2014). The R. Saint John hatchery strain showed a reverse pattern, in that the domesticated strain harbored more diversity than the wild one. It is possible that the original hatchery stock was established from individuals collected from multiple locations or that the effective population size of the wild population decreased following the establishment of the domesticated strain. However, although the loss of diversity in the initial phases of domestication appears to be small, it may become a concern in the long-term maintenance of hatchery populations. To compensate for the loss of genetic diversity, supplementing hatchery stocks with individuals of wild origin may be beneficial.

The understanding of the genetic basis of economically important traits in aquaculture species could provide background information when establishing marker-assisted selective (MAS) breeding programs. Although several QTL studies have been conducted in aquaculture species to identify loci underlying traits such as meat quality, disease resistance, and reproduction, few studies have been able to identify the causative loci (Yue 2013). One such example is the identification of a major locus affecting resistance to infectious pancreatic necrosis (IPN) in Atlantic salmon. MAS programs taking into account the allelic variation in this IPN locus have been initiated to establish IPN-resistant salmon strains (Moen et al. 2009). The challenge in MAS is to identify all loci affecting the trait of interest, including those with small effects. In this respect, our study indicates that the identification of loci with small effects in Atlantic salmon and in other aquaculture species with similar domestication/captive breeding history may be challenging.

## Conclusions

We found little evidence for signals of domestication/inadvertent selection or inbreeding depression in the investigated domesticated/captive breeding strains. Only a slight loss of genetic diversity in domesticated strains was observed, and only a small fraction of loci appear to be under selection. However, the short time interval since the initiation of the hatchery stocks, the possible polygenic background of the quantitative traits, and that the selection appears to be operating on standing genetic variation may all contribute to the low prevalence or inconsistent footprints of selection. In other words, salmon domestication may not involve classical selective sweeps with strong selection on loci with large effects driving beneficial alleles to fixation, but instead subtle shifts in allele frequencies in QTLs underlying adaptation to the hatchery environment. Therefore, if selection is operating in recently domesticated strains, it may be difficult to detect using standard outlier statistics. It is also possible that the coverage of SNPs was insufficient and that some signals of selection were missed. We suggest that future studies in the same framework should employ a denser set of markers genotyped in a larger number of strains with different captive breeding backgrounds.

## Data archiving statement

Data for this study are available at Dryad Digital Repository: http://doi.org/10.5061/dryad.5p7s0.

## Supporting Information

**Figure S1.**LD heat maps of the linkage groups containing outlier loci. The positions of the outlier loci are indicated with blue asterisks.

**Figure S2.**LD decay plots for each population showing the genome-wide decay of LD as measured by *r*^2^ and D'.

**Figure S3.**SNP density in the three domesticated/wild comparisons. The densities are plotted in approximately 1 Mb bins. Note the large gaps especially in R. Saint John.
