# Supplementary material for: Population genomic analyses of early-phase Atlantic Salmon (Salmo salar) domestication/captive breeding
Source: Evol Appl. 2014 Nov 20;8(1):93–107. doi: 10.1111/eva.12230 (PMC4310584; doi:10.1111/eva.12230)
Supplement: Supplementary file 1 [file eva0008-0093-sd1.pdf]

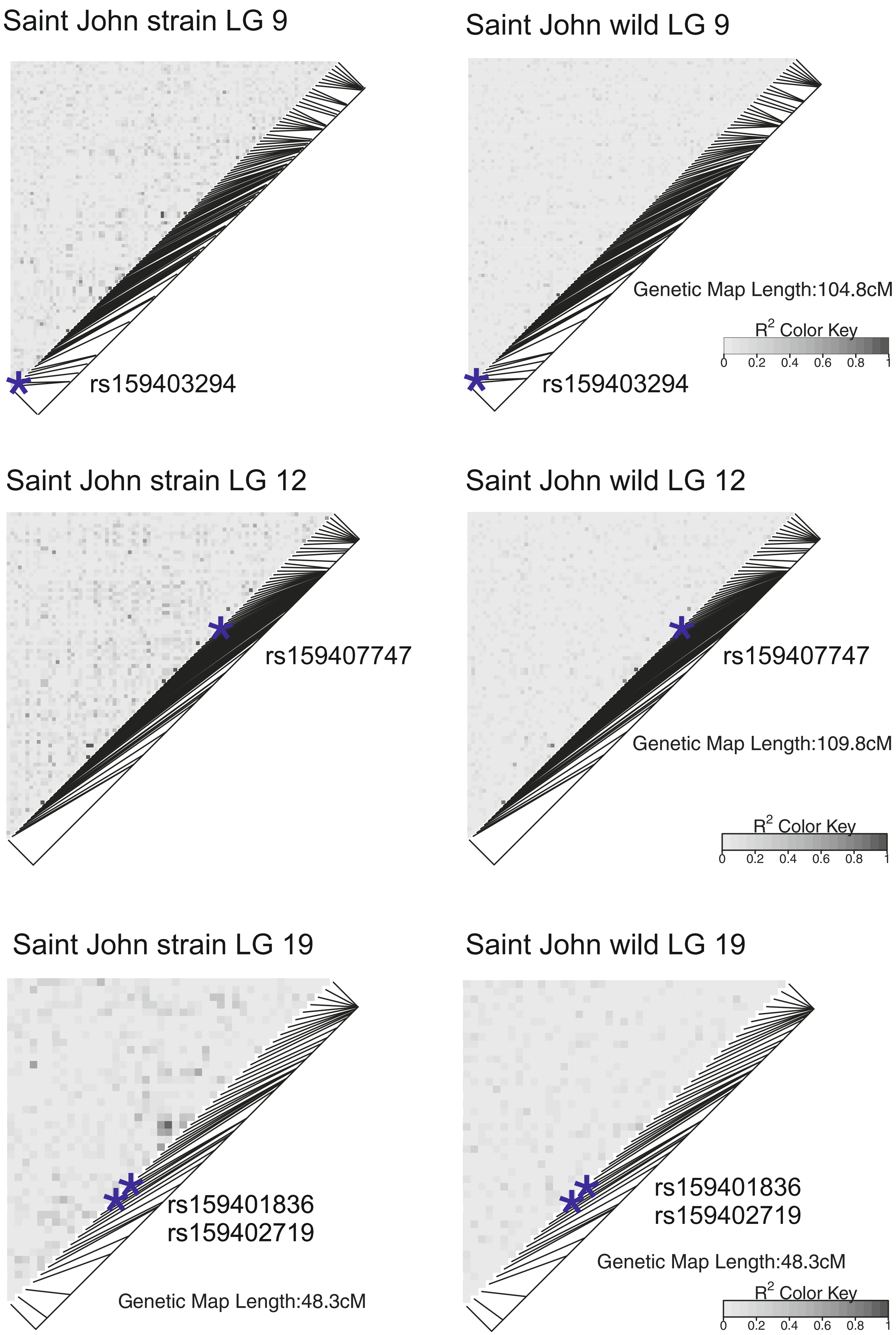

Saint John strain LG 9
rs159403294
Saint John wild LG 9
Genetic Map Length:104.8cM
R2 Color Key
0 0.2 0.4 0.6 0.8 1
rs159403294
Saint John strain LG 12
rs159407747
Saint John wild LG 12
rs159407747
Genetic Map Length:109.8cM
R2 Color Key
0 0.2 0.4 0.6 0.8 1
Saint John strain LG 19
rs159401836
rs159402719
Genetic Map Length:48.3cM
Saint John wild LG 19
rs159401836
rs159402719
Genetic Map Length:48.3cM
R2 Color Key
0 0.2 0.4 0.6 0.8 1

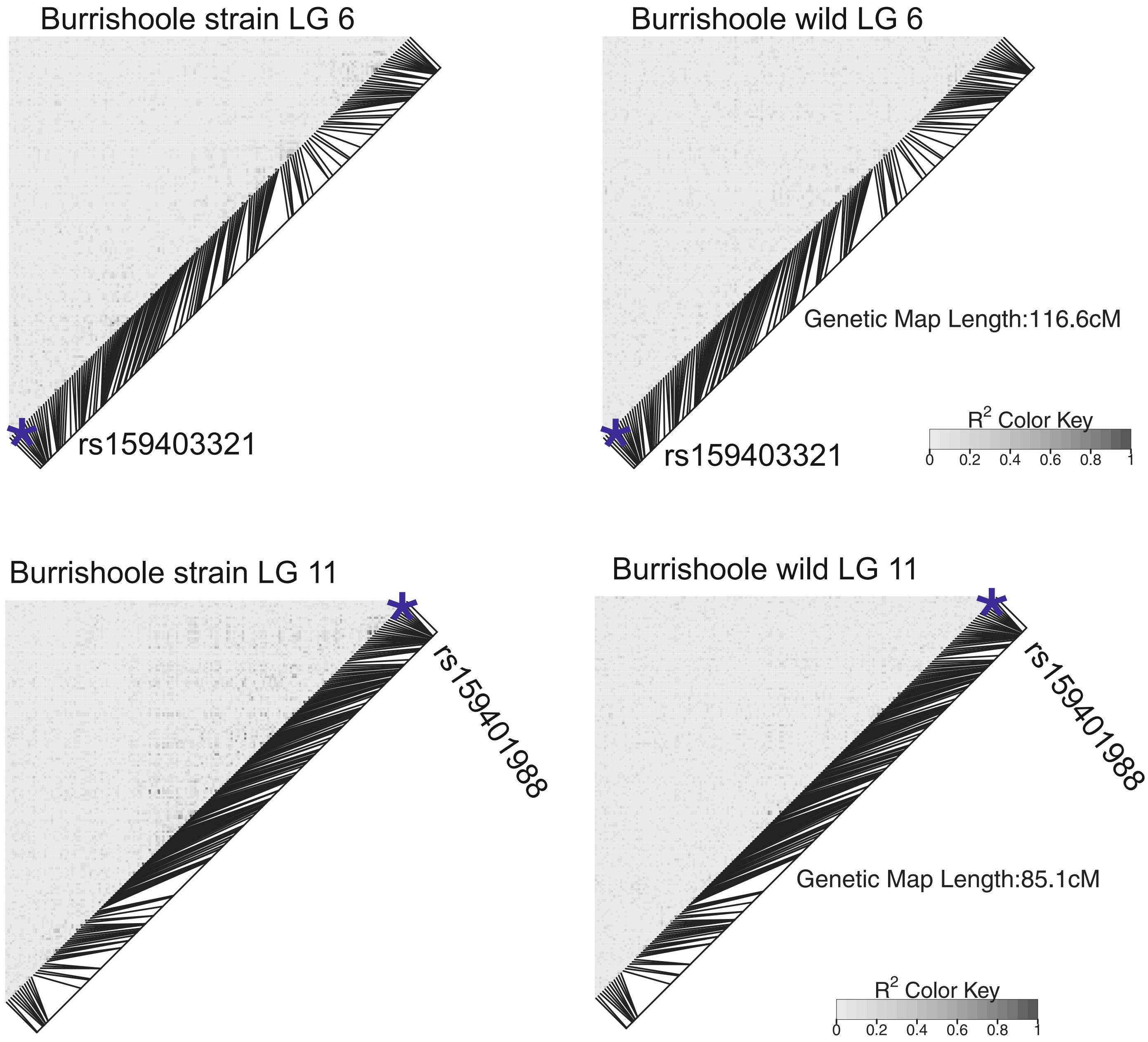
Burrishoole strain LG 6
rs159403321
Burrishoole wild LG 6
Genetic Map Length:116.6cM
rs159403321
R2 Color Key
0 0.2 0.4 0.6 0.8 1
Burrishoole strain LG 11
rs159401988
Burrishoole wild LG 11
rs159401988
Genetic Map Length:85.1cM
R2 Color Key
0 0.2 0.4 0.6 0.8 1
