# Supplementary material for: Population genomic analyses of early-phase Atlantic Salmon (Salmo salar) domestication/captive breeding
Source: Evol Appl. 2014 Nov 20;8(1):93–107. doi: 10.1111/eva.12230 (PMC4310584; doi:10.1111/eva.12230)
Supplement: Supplementary file 2 [file eva0008-0093-sd2.pdf]

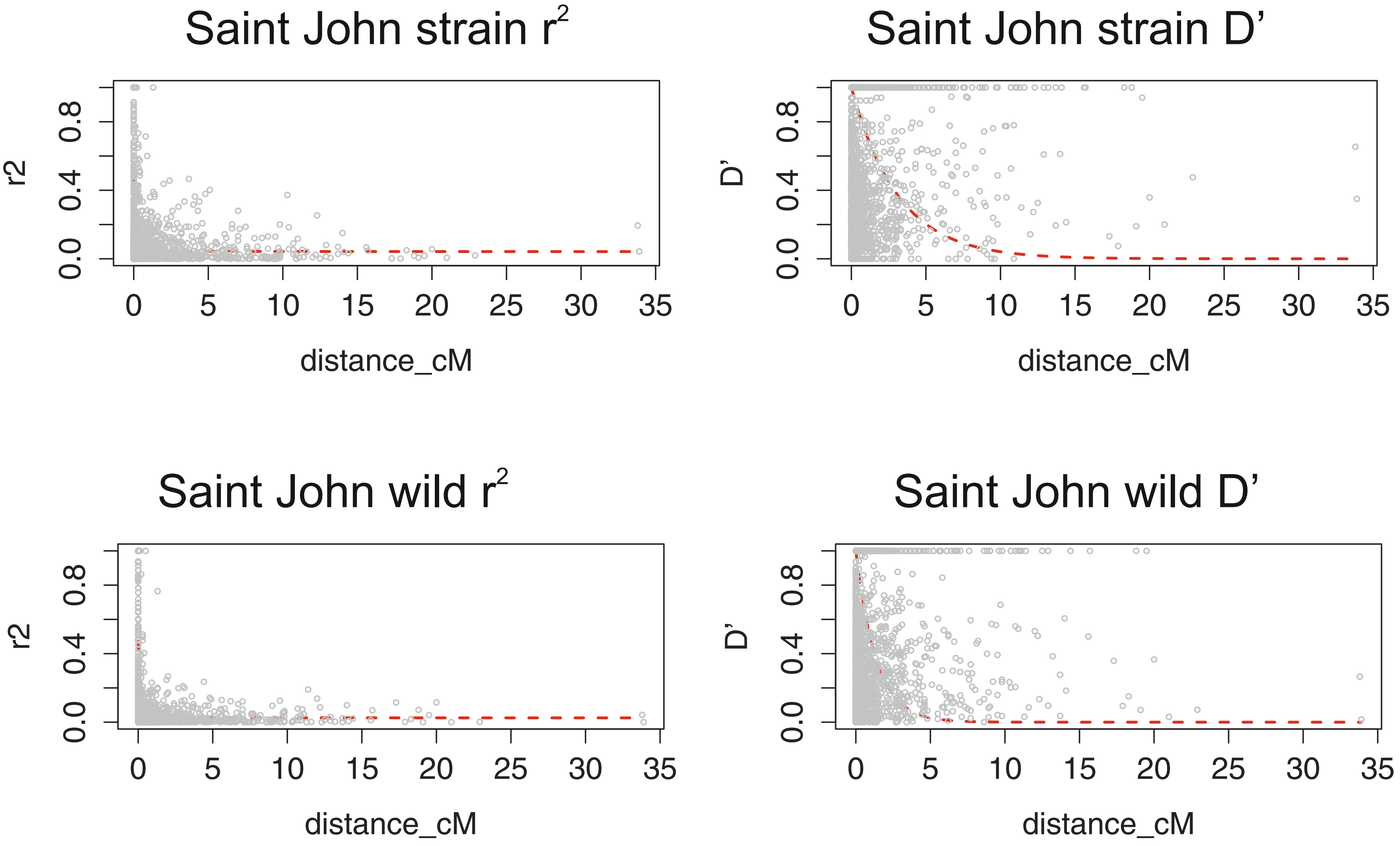

Saint John strain r^2
r2
0.0 0.4 0.8
0 5 10 15 20 25 30 35
distance_cM
Saint John strain D’
D’
0.0 0.4 0.8
0 5 10 15 20 25 30 35
distance_cM
Saint John wild r^2
r2
0.0 0.4 0.8
0 5 10 15 20 25 30 35
distance_cM
Saint John wild D’
D’
0.0 0.4 0.8
0 5 10 15 20 25 30 35
distance_cM

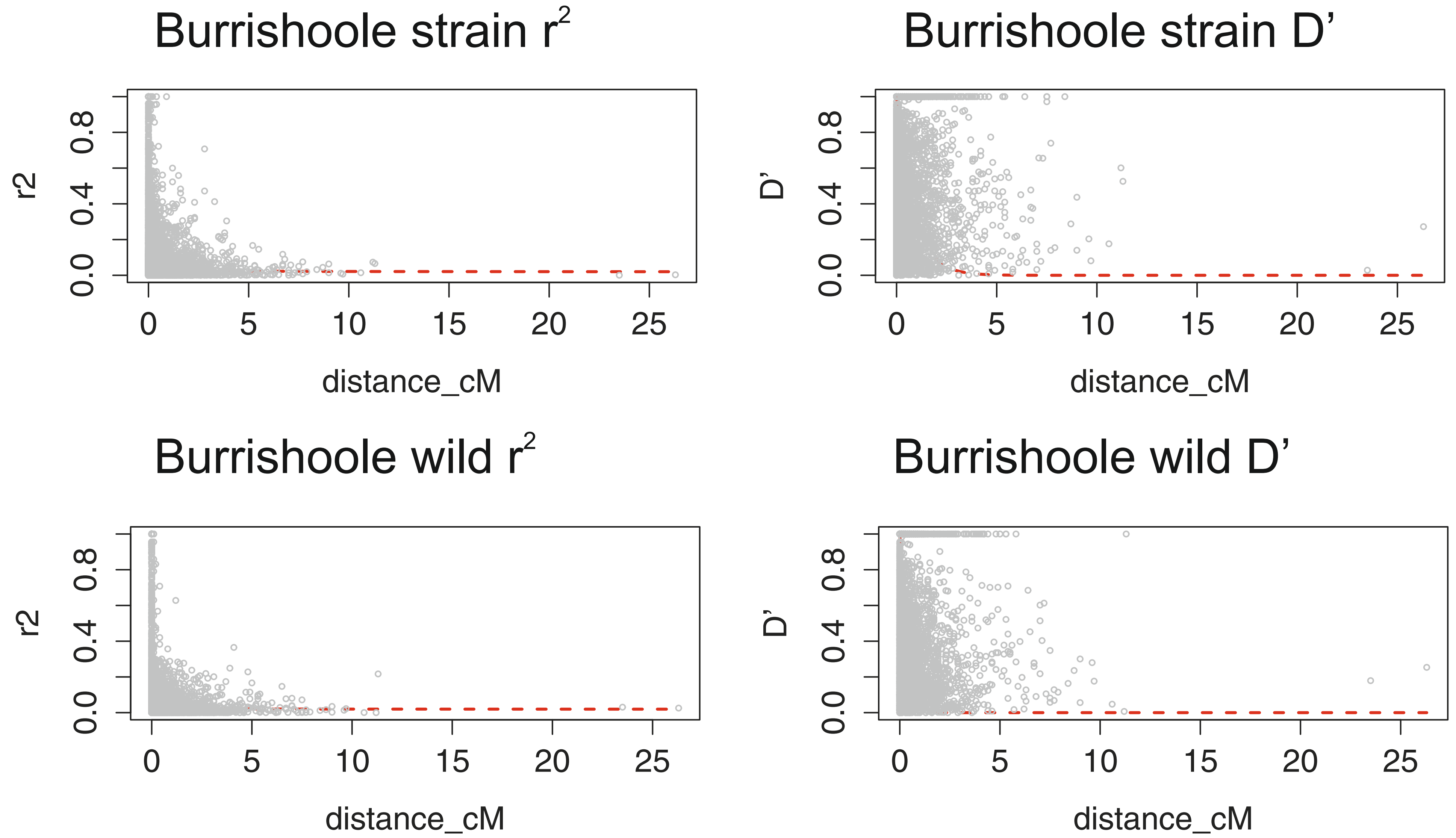
Burrishoole strain r^2
r2
0.0 0.4 0.8
0 5 10 15 20 25
distance_cM
Burrishoole strain D'
D'
0.0 0.4 0.8
0 5 10 15 20 25
distance_cM
Burrishoole wild r^2
r2
0.0 0.4 0.8
0 5 10 15 20 25
distance_cM
Burrishoole wild D'
D'
0.0 0.4 0.8
0 5 10 15 20 25
distance_cM

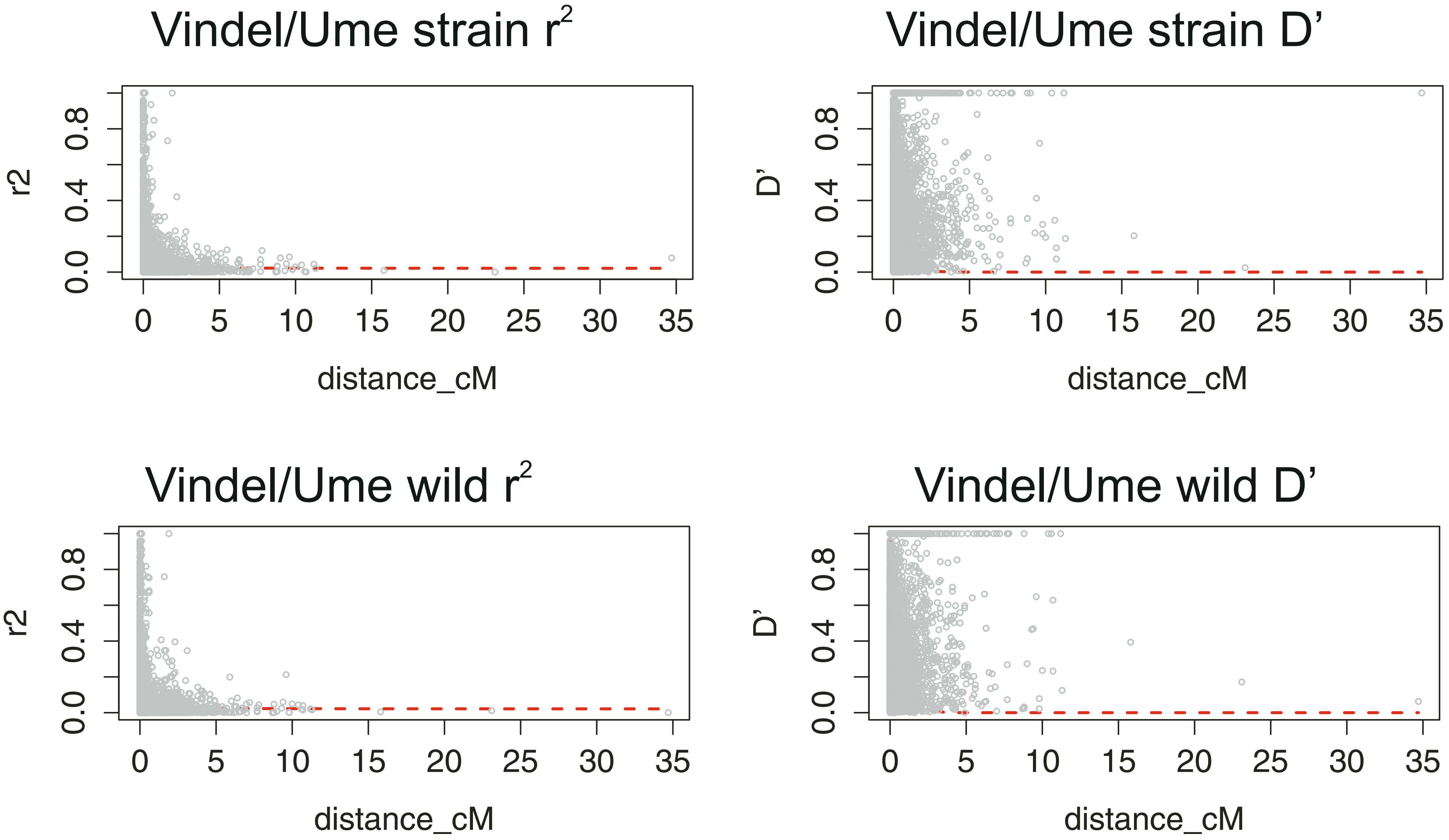

Vindel/Ume strain r2
r2
0.0 0.4 0.8
0 5 10 15 20 25 30 35
distance_cM
Vindel/Ume strain D'
D'
0.0 0.4 0.8
0 5 10 15 20 25 30 35
distance_cM
Vindel/Ume wild r2
r2
0.0 0.4 0.8
0 5 10 15 20 25 30 35
distance_cM
Vindel/Ume wild D'
D'
0.0 0.4 0.8
0 5 10 15 20 25 30 35
distance_cM
