# Supplementary figures and images for: Population genomic analyses of early-phase Atlantic Salmon (Salmo salar) domestication/captive breeding
Source: Evol Appl. 2014 Nov 20;8(1):93–107. doi: 10.1111/eva.12230 (PMC4310584; doi:10.1111/eva.12230)

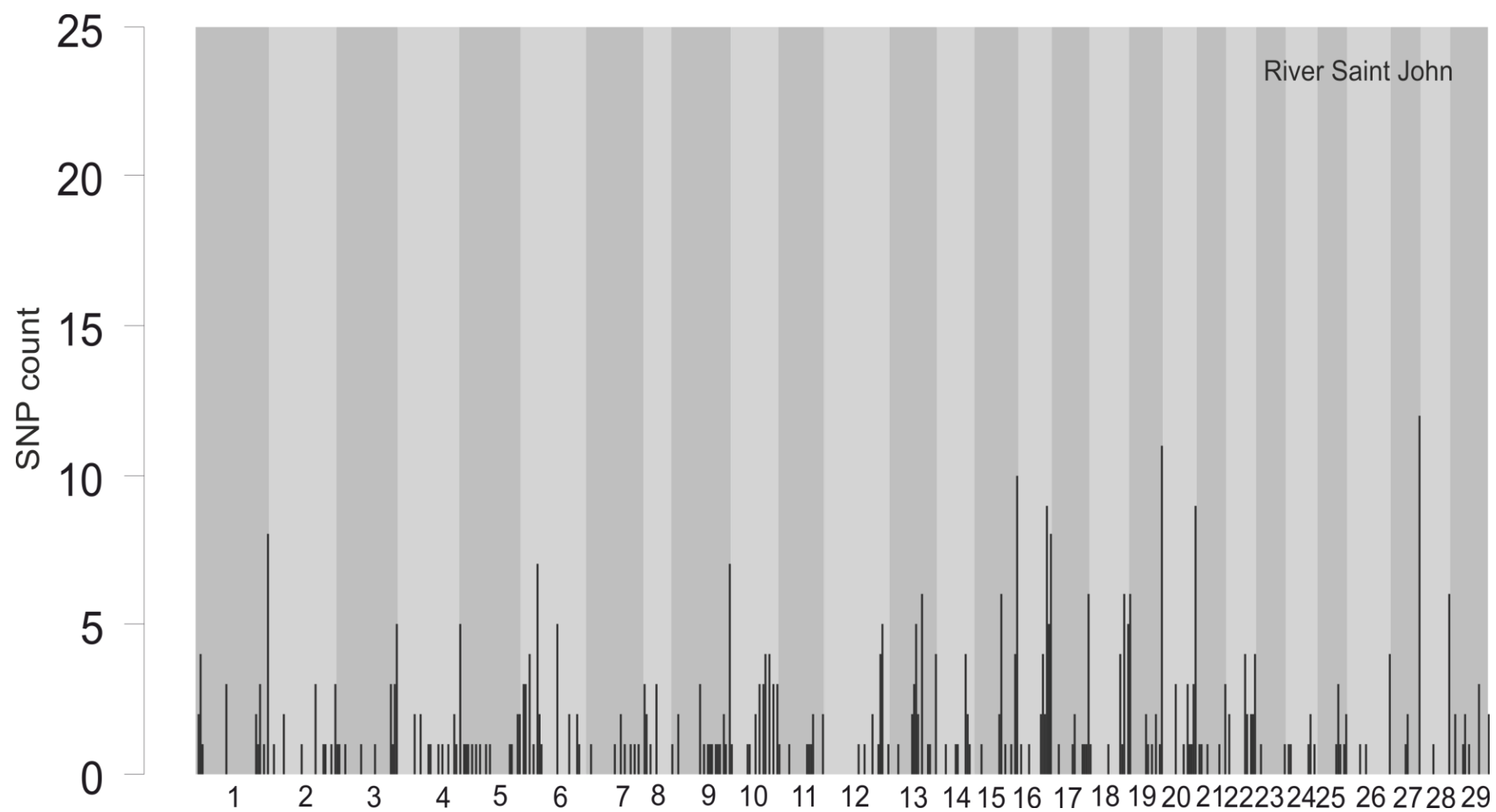

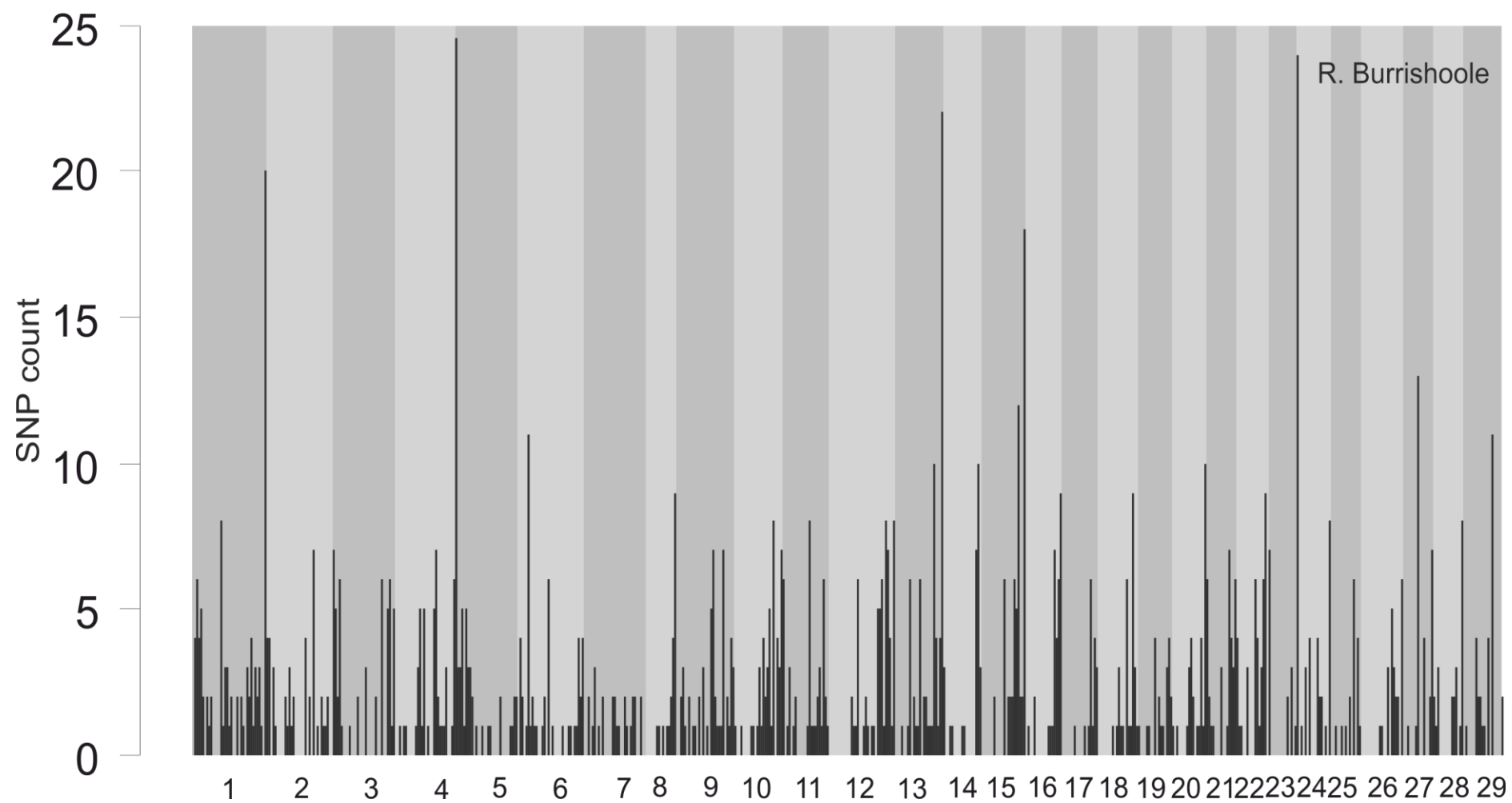

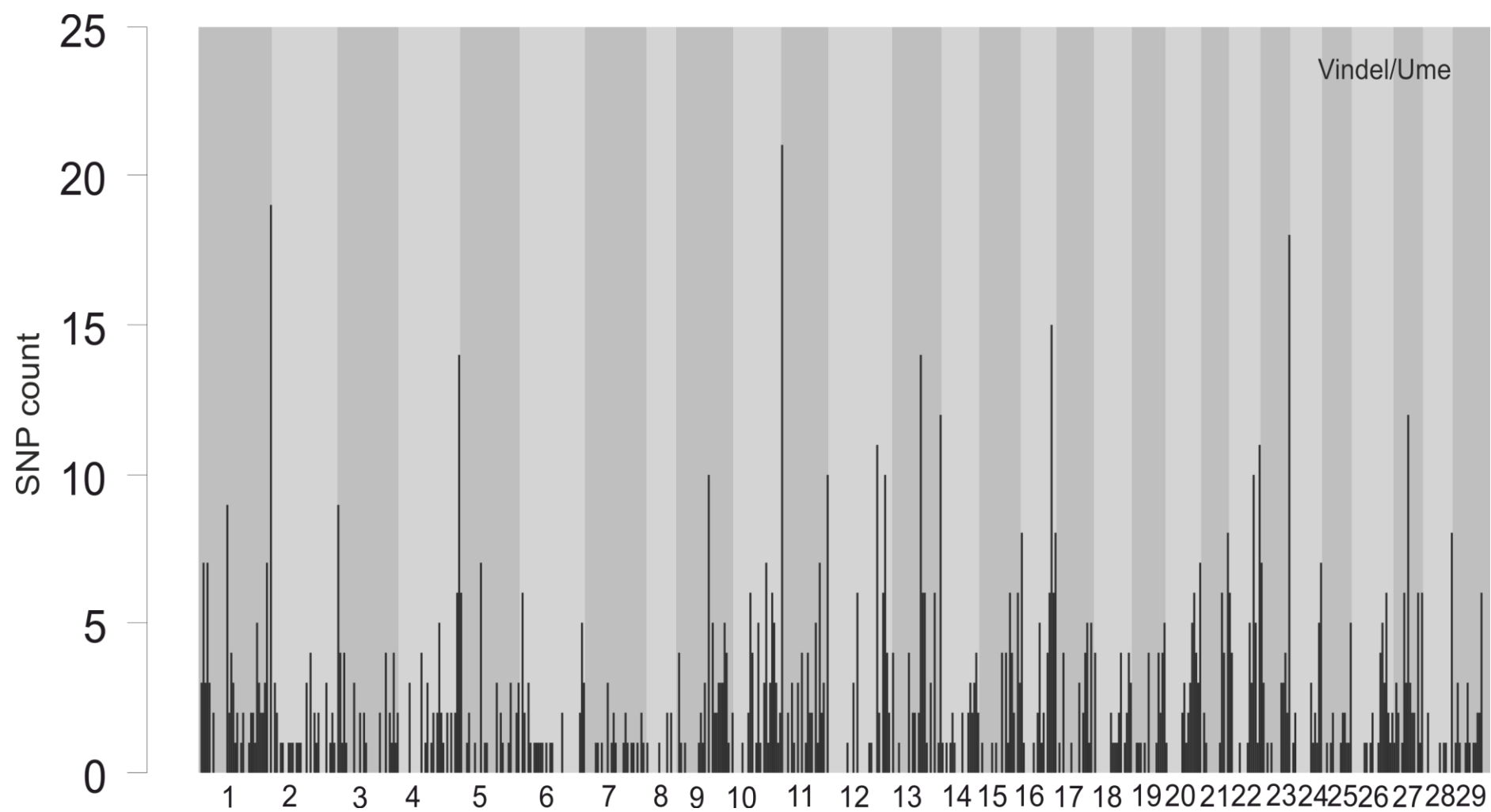

Supplement: Supplementary file 3 [file eva0008-0093-sd3.pdf]
